# Effect of Reduced Graphene Oxide on Microwave Absorbing Properties of Al_1.5_Co_4_Fe_2_Cr High-Entropy Alloys

**DOI:** 10.3390/e26010060

**Published:** 2024-01-10

**Authors:** Shuo Wang, Weiran Zhang, Yong Zhang, Jinqiang Zhao, Ruixuan Li, Yujie Zhong

**Affiliations:** 1 School of Materials Science and Engineering, Xi’an Shiyou University, Xi’an 710065, China; ws571981756@163.com (S.W.); zjq1274031114@163.com (J.Z.); 2 Xi’an Rare Metal Materials Institute Co., Ltd., Xi’an 710016, China; 3 State Key Laboratory for Advanced Metals and Materials, Beijing Key Laboratory for Magneto-Photoelectrical Composite and Interface Science, University of Science and Technology Beijing, Beijing 100083, China; liruixuan1208@163.com

**Keywords:** high-entropy alloys, reduced graphene oxide, microwave absorber, reflection loss, effective absorbing bandwidth

## Abstract

The microwave absorption performance of high-entropy alloys (HEAs) can be improved by reducing the reflection coefficient of electromagnetic waves and broadening the absorption frequency band. The present work prepared flaky irregular-shaped Al_1.5_Co_4_Fe_2_Cr and Al_1.5_Co_4_Fe_2_Cr@rGO alloy powders by mechanical alloying (MA) at different rotational speeds. It was found that the addition of trace amounts of reduced graphene oxide (rGO) had a favorable effect on the impedance matching, reflection loss (RL), and effective absorbing bandwidth (EAB) of the Al_1.5_Co_4_Fe_2_Cr@rGO HEA composite powders. The EAB of the alloy powders prepared at 300 rpm increased from 2.58 GHz to 4.62 GHz with the additive, and the RL increased by 2.56 dB. The results showed that the presence of rGO modified the complex dielectric constant of HEA powders, thereby enhancing their dielectric loss capability. Additionally, the presence of lamellar rGO intensified the interfacial reflections within the absorber, facilitating the dissipation of electromagnetic waves. The effect of the ball milling speed on the defect concentration of the alloy powders also affected its wave absorption performance. The samples prepared at 350 rpm had the best wave absorption performance, with an RL of −16.23 and −17.28 dB for a thickness of 1.6 mm and EAB of 5.77 GHz and 5.43 GHz, respectively.

## 1. Introduction

The rapid development of information technology has increased the complexity and variability of the modern warfare environment [1], and radar detection has become the main means of capturing information and detecting objects in modern information electronic warfare [2,3]. In regard to achieving long-distance camouflage and concealment of weapons and equipment, electromagnetic wave absorbing materials that offer protection against radar detection play an increasingly important role [4]. These materials can substantially absorb and weaken electromagnetic energy and convert it into thermal energy as an effective solution against electromagnetic radiation [5]. Ideally, new absorbing materials for practical applications must have the following characteristics: “thin thickness, light mass, broadband, and strong absorption” to enable them to function in complex environments [6].

Dielectric and magnetic losses are known components of the microwave loss mechanism, and effective complementarity between these components is required to produce excellent microwave absorbing materials [7]. In general, it is difficult for HEA absorbing materials to have excellent dielectric loss capability and magnetic loss capability at the same time. Therefore, it is an effective way to improve the microwave absorbing properties of materials by compounding two materials together to improve the dielectric or magnetic loss capacity. By now, the research of carbon-based magnetic composites has made great progress, and the absorbers prepared by combining ferromagnetic materials and one-dimensional carbon materials (carbon fiber, carbon nanotubes, and biomass-derived carbon) have shown better absorption performance than single absorbers [8]. rGO is a new carbon material that also has the potential for electromagnetic absorption. Wang et al. [9] found that, in addition to enhancing impedance matching characteristics, residual defects and groups also promoted the transition of adjacent states to the Fermi level and polarization relaxation, thus promoting the penetration and absorption of electromagnetic waves. In addition, the two-dimensional structure of rGO has excellent physical properties, such as being lightweight and having a large specific surface area, and having good thermal conductivity, all of which are conducive to its application as a wave absorbing material. However, graphene has problems such as poor dispersion [10], interface impedance mismatch [11], and a single loss mechanism in the matrix. Therefore, it is necessary to combine other materials capable of electromagnetic absorption into rGO for coupling to improve the wave absorbing performance. Chen [12] prepared a composite material consisting of rGO and carbonyl iron powders using the MA method, which optimized the dielectric and magnetic losses of the carbonyl iron powders and significantly improved the wave absorption ability. RL reached −32.3 dB at a thickness of 2.0 mm for 50 wt% of rGO/FCI/epoxy absorber. Liu [13] investigated the wave absorption performance of single- and double-layer absorbers comprising Co_0.2_Ni_0.4_Zn_0.4_Fe_2_O_4_ and rGO composites and showed that the maximum RL of the 2.5 mm double-layer absorber at 16.9 GHz reached −49.5 dB with an effective bandwidth as high as 6.0 GHz below −10 dB. The rGO layer was shown to have strong dielectric loss ability. Ding [14] synthesized CuFe_2_O_4_/rGO composites, and the residual defects of rGO and defect polarization caused by oxygen-containing groups improved the microwave absorption. EAB reached 5.2 GHz at a thickness of 1.85 mm, and RL at 9.2 GHz at a thickness of 2.56 mm was −58.7 dB. These previous studies showed that rGO doping can effectively improve the dielectric loss of ferromagnetic metal absorbing materials and obtain better microwave absorption performance.

Recently, HEAs have gained wide attention in the field of electromagnetic wave absorption due to their excellent corrosion, high temperature, and oxidation resistance. There are a variety of processing routes for the synthesis of HEAs, and MA is widely used because of its high efficiency and cost-effectiveness. It can effectively achieve uniform mixing of multiple metal elements, thus obtaining a more uniform HEA organizational structure and excellent performance [15]. Duan [16,17] prepared FeCoNiSi_x_Al and FeCoNiCu_x_Al HEA powders by MA and studied the electromagnetic properties of the powders. The results showed that the HEA had good soft magnetic properties and electrical conductivity, which are highly potential wave absorbing materials. Yang [18] effectively regulated the electromagnetic properties and improved the electromagnetic wave absorbing properties by doping C into FeCoNiCu HEA powders. Wang et al. [19] used a cluster-based compositional design approach to obtain Al_1.5_Co_4_Fe_2_Cr HEAs, and the results showed that these HEAs had excellent soft magnetic properties, with a high saturation magnetization (Ms = 135.3 emu/g) and a low coercivity force (H_C_ = 127.3 Oe). However, its electromagnetic absorbing ability was not explored. Moreover, Tan [20] prepared FeCoNiAlx alloys with close ferromagnetic element contents, and the comparison revealed that the BCC phase had a higher M_S_ compared with the FCC phase. Therefore, in this paper, Al_1.5_Co_4_Fe_2_Cr HEA powders with BCC phase were prepared by MA, and their electromagnetic wave absorbing abilities were investigated.

Al_1.5_Co_4_Fe_2_Cr HEA powders with rGO were prepared by MA at ball milling speeds of 300, 350, and 400 rpm, respectively. The effects of the ball milling speed and doping rGO on the electromagnetic absorption ability of Al_1.5_Co_4_Fe_2_Cr were systematically studied. The experimental results show that the doping of rGO was helpful in improving the impedance-matching characteristics and dielectric loss of the alloy powder. In addition, the higher milling speed made the powder more strongly impacted by the stainless-steel grinding ball, which produced more defects and impurities, and changed the complex permittivity and permeability of the material, which ultimately enabled the material to effectively absorb and attenuate electromagnetic waves.

## 2. Materials and Methods

**Figure 1:**
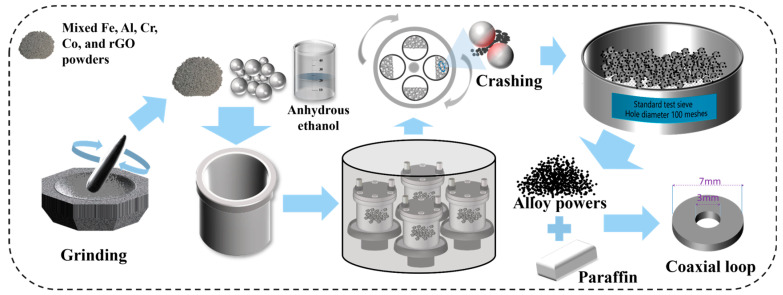
Schematic diagram of the preparation process.

The fabrication process of Al_1.5_Co_4_Fe_2_Cr and Al_1.5_Co_4_Fe_2_Cr@rGO HEA powders is shown in Figure 1. High-purity (>99 wt.%, weight percent) Fe, Co, Al, Cr, and rGO in powder form (particle size < 50 µm) were used as raw materials, and three kinds of steel balls of 10 mm, 8 mm, and 5 mm were used as the milling medium, with the mass ratio of 5:1:4. The mixed raw materials (30 g) were ground for 30 min, placed in a vacuum ball-milling tank with 20 mL of absolute ethanol and 600 g of stainless steel grinding balls, sealed and vacuumed. High-energy ball milling was performed using an omnidirectional planetary ball mill (QM-3SP4), and the ball milling speeds were set to 300, 350, and 400 rpm, respectively. Intermittent milling was applied, viz. milling for 30 min and then suspending for 4 min. Each sample was milled for a total of 100 h. After the first ball mill, the powders were dried at 60 °C for 12 h under vacuum, and then ball milled for 30 min at a speed of 200 rpm. The stainless-steel grinding balls were separated from the powders using a 100 mesh standard inspection sieve to obtain HEA powders. The powder samples were denoted as A_300_, A_350_, and A_400_ (Al_1.5_Co_4_Fe_2_Cr) and B_300_, B_350_, and B_400_ (Al_1.5_Co_4_Fe_2_Cr@rGO), where the subscripts represent the ball milling speed at which the powder samples were prepared.

The microstructures of the alloy powder samples were characterized with the aid of X-ray diffraction (XRD, MinFlex600/600-C) in the 2θ scan range from 20° to 90° at the operating voltage and tube current of 40 kV and 30 mA. The degree of defects and molecular structure of the prepared powder samples were analyzed using a Raman spectrometer (Horiba Scientific LabRAM HR Evolution). The microscopic morphology and element distribution of the powders were performed by a scanning electron microscope (SEM, EM30AX+) with an energy dispersive spectrometer (EDS) detector. The particle size distribution was measured using a laser-scattering particle size distribution analyzer. A vibrating sample magnetometer (VSM, MPMS-3) was employed to investigate the magnetic properties, such as M_S_ and H_C_, at a maximum applied field of 20,000 Oe at room temperature. The alloy powders were uniformly mixed with paraffin wax at a weight ratio of 7:3 to form a concentric ring with an inner diameter of 3.0 mm, an outer diameter of 7.0 mm, and a thickness of 2.0 mm. The complex permittivity and complex permeability of the powder samples were measured in the frequency range of 2–18 GHz using a vector network analyzer (Agilent E5071C) via a coaxial method.

## 3. Results and Discussion

### 3.1. Microstructure of Al_1.5_Co_4_Fe_2_Cr and Al_1.5_Co_4_Fe_2_Cr@rGO HEA Powders

**Figure 2:**
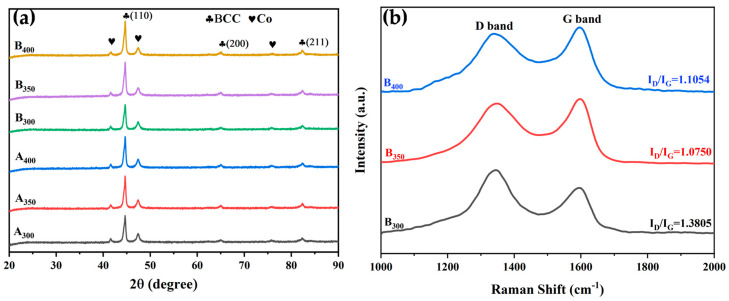
(**a**) XRD patterns of Al_1.5_Co_4_Fe_2_Cr and Al_1.5_Co_4_Fe_2_Cr@rGO HEA powders prepared at different milling speeds and (**b**) Raman patterns of Al_1.5_Co_4_Fe_2_Cr@rGO HEA powders.

Figure 2a shows the XRD patterns and crystal structure of Al_1.5_Co_4_Fe_2_Cr and Al_1.5_Co_4_Fe_2_Cr@rGO alloy powder samples prepared by a high-energy ball milling process at three different milling speeds. The cycle of cold welding and crushing during ball milling promoted the mutual dissolution of different elements [21]. These five elements were not fully alloyed, instead forming solid solutions with a simple crystal structure in the BCC phase (identified by peaks corresponding to the (110), (200), and (211)) and a small percentage of residual undissolved Co atoms. This was mainly due to the large atomic size difference between Co and other metal elements and its high content, leading to incomplete dissolution. In addition, no characteristic rGO peaks were observed for B_300_, B_350_, or B_400_, possibly due to their weak carbon peaks being overwhelmed by strong signals of the alloys.

The microstructure of the Al_1.5_Co_4_Fe_2_Cr@rGO HEA powders was characterized by Raman spectroscopy. Figure 2b shows the Raman spectra of Al_1.5_Co_4_Fe_2_Cr@rGO powder samples prepared at different speeds in the range of 1000 to 2000 cm^−1^. The two distinct peaks near 1350 cm^−1^ and 1600 cm^−1^ were the D-band and G-band peaks of rGO, respectively. The D-band peaks were generally the result of disordered carbon and other defects at the edge, while the G-band peaks corresponded to the in-plane tensile vibration of the sp^2^ hybridized C atoms and were the dominant characteristic peaks of graphene materials [22]. The density of lattice defects in carbon materials is typically characterized by the intensity ratio I_D_/I_G_. The larger the I_D_/I_G_ value, the greater the number of lattice defects [23]. The I_D_/I_G_ values corresponding to the rotational speeds of 300, 350, and 400 rpm were 1.38, 1.075, and 1.105, respectively. The I_D_/I_G_ ratio of the sample prepared at 300 rpm was significantly higher than that of the samples prepared at 350 and 400 rpm. This indicates that the sample prepared at 300 rpm had greater disorder and more lattice defects. In contrast, the higher milling speed increased the stress experienced by the rGO, thereby decreasing the average crystal domain size to form more sp^2^ hybrid domains. This increased the intensity of the G peak and lowered the I_D_/I_G_ ratio.

**Figure 3:**
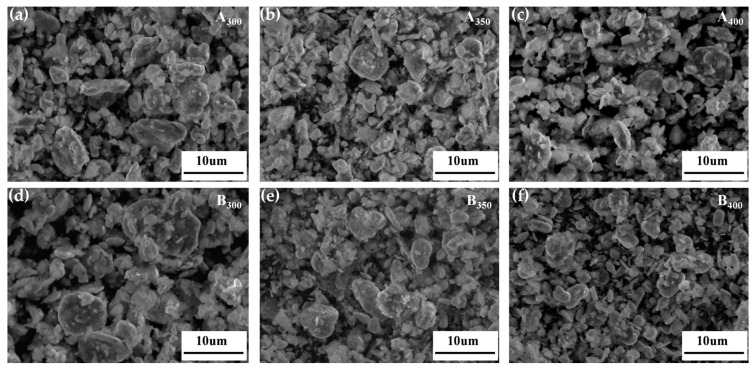
SEM images of Al_1.5_Co_4_Fe_2_Cr and Al_1.5_Co_4_Fe_2_Cr@rGO HEA powders prepared at different speeds. (**a**) A_300_, (**b**) A_350_, (**c**) A_400_, (**d**) B_300_, (**e**) B_350_, and (**f**) B_400_.

**Figure 4:**
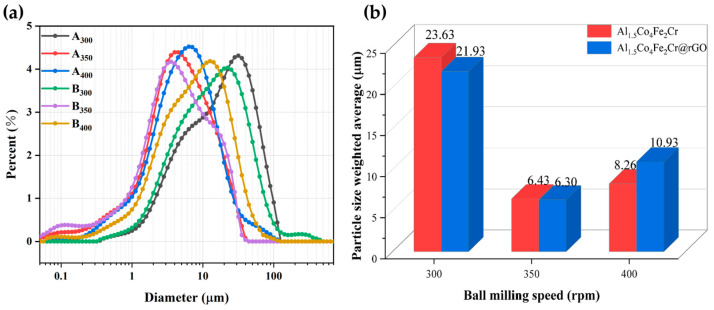
(**a**) Particle size distributions of Al_1.5_Co_4_Fe_2_Cr and Al_1.5_Co_4_Fe_2_Cr@rGO HEA powders prepared at different speeds; (**b**) particle size weighted average.

Figure 3 shows the SEM images of the Al_1.5_Co_4_Fe_2_Cr and Al_1.5_Co_4_Fe_2_Cr@rGO powder samples prepared at different rotational speeds. The particles in the powder samples had the shapes of irregular flakes, with particle sizes ranging from a few micrometers to several hundred nanometers. According to previous studies, sheet materials with greater anisotropy in shape will be more favorable for electromagnetic wave absorption [24,25]. The coexistence of fragmentation and cold welding during the ball milling process reduced the size of some of the flakes, which were then pressed together to form a rough surface. The enhanced surface roughness was more conducive to multiple reflections and the absorption of electromagnetic waves. With the increased rotational speed, the stronger kinetic energy broke the HEAs powder into smaller flake particles, which can be confirmed by the particle size distribution in Figure 4a,b. The average particle size of Al_1.5_Co_4_Fe_2_Cr HEA powders decreased from 23.63 to 6.43 um with the increase in milling speed from 300 rpm to 350 rpm. However, after subsequently increasing the rotational speed to 400 rpm, the average particle size of the alloy powders increased to 8–11 um.

**Figure 5:**
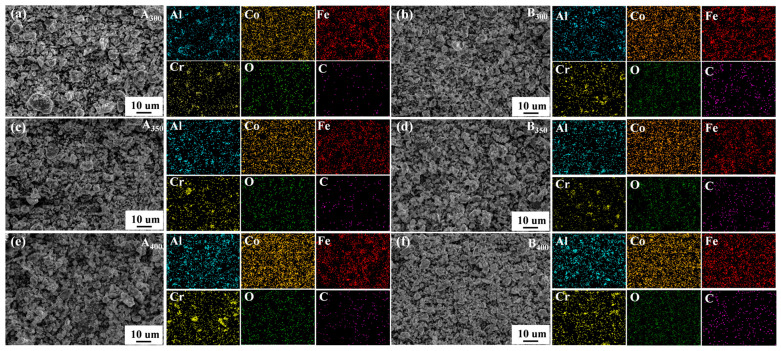
Elements mapping of Al_1.5_Co_4_Fe_2_Cr and Al_1.5_Co_4_Fe_2_Cr@rGO HEA powders. (**a**) A_300_, (**b**) B_300_, (**c**) A_350_, (**d**) B_350_, (**e**) A_400_, and (**f**) B_400_.

The element distribution of samples is shown in Figure 5a–f. It was found that the four component elements (Al, Co, Fe, and Cr) were relatively uniformly dispersed on the samples without significant segregation. In the process of sample preparation, oxygen was inevitably contacted, so the O element was found in the elemental distribution mapping. Remarkably, C element was also found in the samples without added rGO, suggesting the presence of carbon contamination in the powders. This may have been due to the decomposition of anhydrous ethanol into carbides distributed in the powders during ball milling (a similar situation occurred in the study of Duan et al. [26,27]). In addition, relevant chemical composition information of samples that were characterized by EDS is listed in Table 1. It can be seen that the increase in rotational speed increased the level of C contamination in the samples.

### 3.2. Magnetic Properties

**Figure 6:**
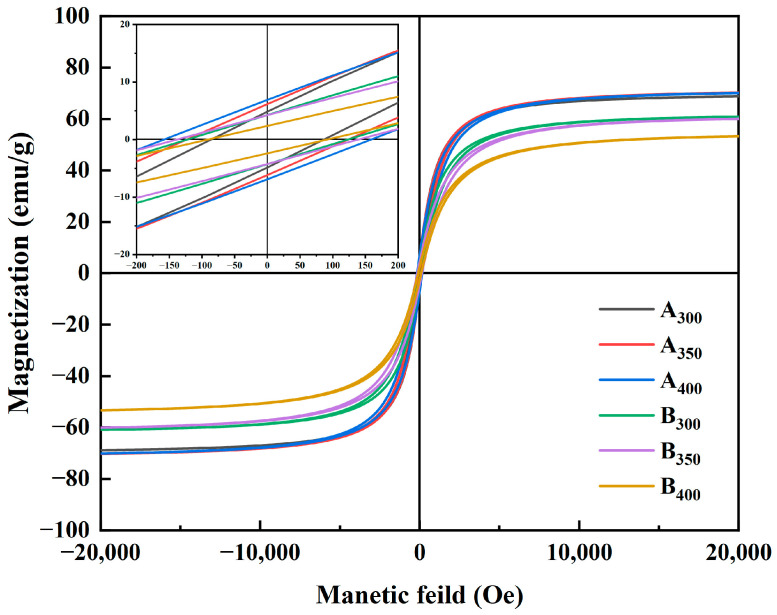
Hysteresis line plots of Al_1.5_Co_4_Fe_2_Cr and Al_1.5_Co_4_Fe_2_Cr@rGO HEA powders prepared at different rotational speeds.

**Figure 7:**
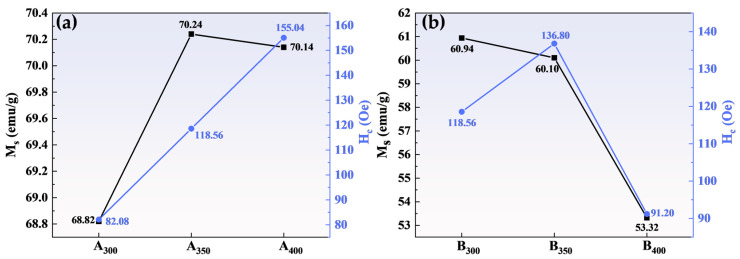
Variation in the M_S_ and coercivity H_C_ of (**a**) Al_1.5_Co_4_Fe_2_Cr and (**b**) Al_1.5_Co_4_Fe_2_Cr@rGO HEA powders prepared at different ball milling speeds.

Figure 6 shows the magnetic hysteresis loops of Al_1.5_Co_4_Fe_2_Cr and Al_1.5_Co_4_Fe_2_Cr@rGO powder samples prepared at different rotational speeds in the presence of an applied magnetic field (±20,000 Oe). All the samples presented a rather narrow hysteresis loop with high M_S_ and low Hc, which was consistent with the properties of soft magnetic materials. Figure 7 depicts in detail the relationship between ball milling speed, rGO, and M_S_ intensity versus H_C_ of HEAs. Because of the weak magnetism of rGO, M_S_ of the three samples decreased after adding rGO, and M_S_ of B_400_ was 16.82 emu/g lower than that of A_400_. M_S_ of Al_1.5_Co_4_Fe_2_Cr increased slightly at a higher rotational speed. However, H_C_ exhibited an upward trend, attaining 82.08 Oe at 300 rpm and 155.04 Oe at 400 rpm. For Al_1.5_Co_4_Fe_2_Cr@rGO, M_S_ of the powder samples continued to decrease with increasing speed from 300 to 400 rpm, reduced from 60.94 to 53.32 emu/g. Hc increased at first and then decreased with the decrease in rotational speed. When the rotational speed increased from 350 rpm to 400 rpm, Hc decreased from 136.80 to 91.20.

The M_S_ of a material is primarily determined by the composition and microstructure of the material, whereas the coercivity is primarily affected by the grain size and impurities [28]. As shown in Figure 7a, the increase in rotational speed had little effect on M_S_ but caused the Al_1.5_Co_4_Fe_2_Cr HEA powders to collide more powerfully with the sphere during ball milling, which provided plenty of defects and thus enhanced H_C_. As to M_S_, as shown in Figure 7b, although the microstructure was not significantly altered, the increase in non-magnetic components decreased the magnetization of Al_1.5_Co_4_Fe_2_Cr HEAs. Doping with rGO also led to an increase in defect concentration, resulting in an increase in H_C_ of B_300_ and B_350_.

### 3.3. Electromagnetic Parameters

**Figure 8:**
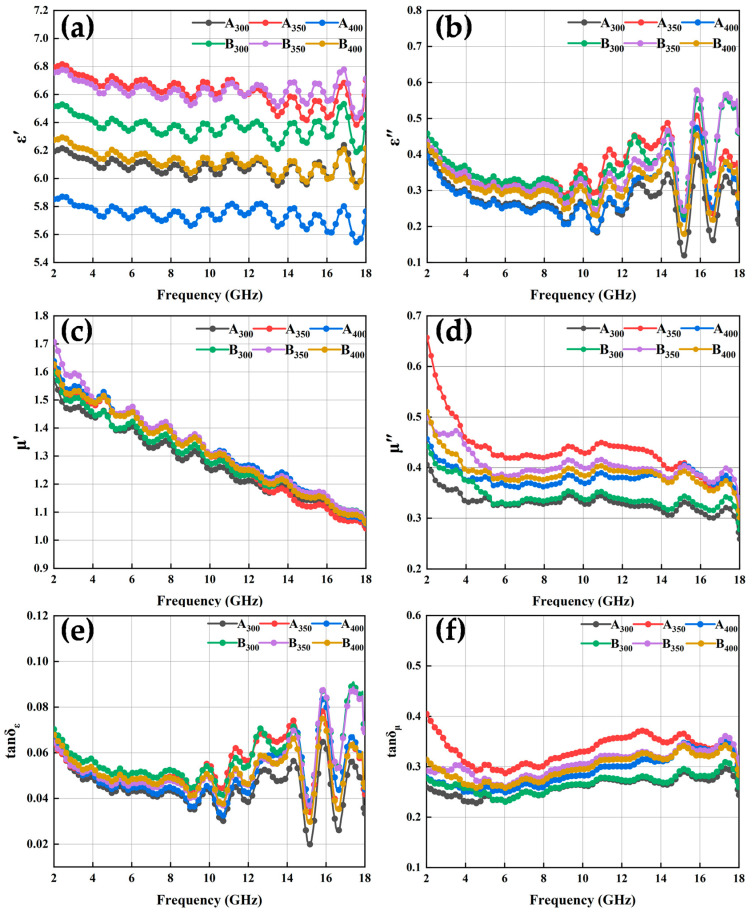
Complex permittivity, complex permeability, and loss tangent diagrams of Al_1.5_Co_4_Fe_2_Cr and Al_1.5_Co_4_Fe_2_Cr@rGO HEA powders with different rotational speeds: (**a**) real (ε′) and (**b**) imaginary (ε″) parts of the composite permittivity; (**c**) real (μ′) and (**d**) imaginary (μ″) parts of the complex permeability; (**e**) the tangent of the dielectric loss (tan δ_ε_) and (**f**) the tangent of the magnetic loss (tan δ_μ_).

The electromagnetic response of HEA powders in the frequency range of 2–18 GHz, including complex permittivity and permeability, is shown in Figure 8. In general, the real parts of complex permittivity (ε′) and permeability (μ′) are considered to evaluate the capacity of electric and magnetic energy store, respectively. The imaginary parts (ε″ and μ″) quantify electric and magnetic energy dissipation, respectively [29].

The results in Figure 8a show that the real part of the composite dielectric constant of HEA powders increased and then decreased as the speed increased, and the ε′ values of the two powders prepared at 350 rpm were higher than those of the other samples, exhibiting a stronger storage capacity of electron energy. In addition, the conductivity characteristics of rGO contributed to raising the conductivity and polarization processes of the powders to which rGO had been added at the same ball milling speed, and the higher conductivity of the alloy powders increased the ε′ value. This can also be explained using the Debye relaxation theory [30]:

(1) $$\varepsilon^{\prime }=\frac{\sigma }{\omega^{2}{\tau \varepsilon }_{o}}{+\varepsilon }_{\infty }$$

(2) $$\varepsilon^{\prime \prime }=\frac{\sigma }{{\omega \varepsilon }_{o}}$$

where σ is the conductivity, ω is the angular frequency (ω = 2πf), τ is the relaxation time of the dipole, and ε_0_ and ε_∞_ are the dielectric constants at static and infinite frequencies, respectively. A positive correlation exists between ε′ and ε″ and σ, and a negative correlation with ω.

The electrical conductivity of the powders was enhanced by the addition of rGO, which led to larger values of ε′. On the other hand, the addition of rGO powders enhanced the interfacial polarization and defect-excited polarization of the alloy powders, which was also the reason for the increase in the ε′ value of the powders. The increase in frequency was followed by an increase in angular frequency, which led to a decrease in the ε′ value of the powders with increasing frequency. As shown in Figure 8b, the ε″ of the alloy powders exhibited a complex trend with increasing frequency, the variation was smoother in the 2–8 GHz range, while it fluctuated more in the 10–18 GHz frequency range. Comparing the ε″ of the alloy powders, the results show that the addition of rGO helped to increase the dielectric loss capacity of HEAs at the same rotational speed. In addition, the real and imaginary parts of the complex permittivity curves of the powders showed multiple resonance peaks in the frequency range of 2–18 GHz, and the dielectric resonance was generally caused by electron polarization, ion polarization, or dipole polarization. Where electron polarization and ion polarization were not applicable in the frequency range of 2–18 GHz, these dielectric resonances were caused by dipole polarization.

**Figure 9:**
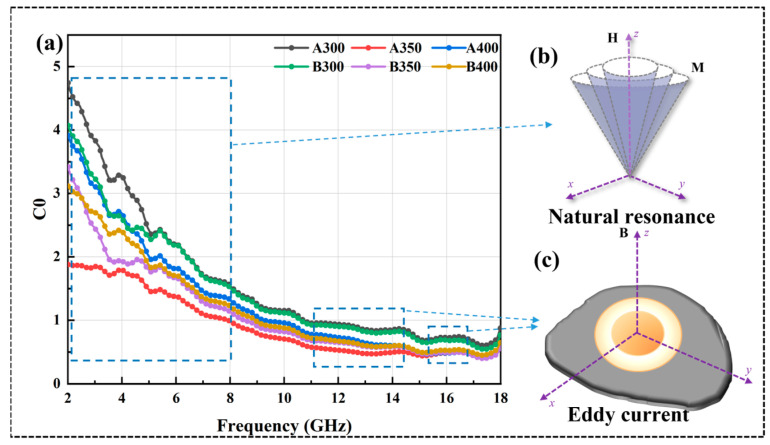
(**a**) C_0_ curves of Al_1.5_Co_4_Fe_2_Cr and Al_1.5_Co_4_Fe_2_Cr@rGO HEA powders, (**b**) natural resonance, and (**c**) eddy current.

The curves of μ′ vs. frequency of all the alloy powder samples (Figure 8c,d) showed a decreasing trend as the frequency increased, which conformed to the law of Snoek’s limit [30]. Similarly, μ″ also exhibited a decreasing trend against the frequency. This could be explained by the initial penetration rate formula [31] and the eddy current formula [17]:

(3) μi ≈ μ0Ms2(K1+32λsσ)β13δd

(4) $$\mu_{r}^{\prime \prime }{=3\pi \mu }_{0}{\left(\mu^{\prime }\right)}^{2}d^{2}f\sigma$$

where M_S_, k_1_, λ_s_, σ, β, δ, and d represent saturation magnetization, magneto crystalline anisotropy constant, magnetostriction coefficient constant, internal strain, impurity volume concentration, domain wall thickness, and impurity diameter, respectively. Because μ″ and μ′ were positively correlated, the curves of μ″ and μ′ showed similar trends. It could be observed that the μ′ value of B_350_ did not decrease due to the decrease in M_S_, which was because the increase in coercivity led to a decrease in k_1_ and λs of the powders, thus increasing the μ′ value. The μ″ value of the powders increased and then decreased with the increase in the ball milling speed, and the μ″ value of the powders prepared at 350 rpm was the largest, which indicated that its magnetic dissipation ability was strong. In addition, the resonance peak appeared in the μ″ curve, which was known to originate from natural resonance by analyzing the C_0_ curve of the material in Figure 9a. Eddy current losses can be characterized as [32]:

(5) $$C_{0}=\mu^{\prime \prime }{\left(\mu^{\prime }\right)}^{-2}f^{-1}$$

When C_0_ tended to be stable, eddy current loss dominated the magnetic loss. With the increase infrequency, the C_0_ curve of all powders gradually tended to stabilize from decreasing; the magnetic loss mode in the range of 2–10 GHz had natural resonance (Figure 9b) and eddy current loss (Figure 9c), and, after 10 GHz, was mainly eddy current loss.

Based on the above complex permittivity and complex permeability, the values of the dielectric and magnetic loss angle tangent of the alloy powder samples were obtained by calculation, as shown in Figure 8e,f. These results show that, in the frequency range of 2–18 GHz, the value of tan δ_ε_ was significantly smaller than that of tan δ_μ_, indicating that the alloy powders absorbed electromagnetic waves mainly via magnetic loss rather than dielectric loss. Notably, the curves of tan δ_ε_ and ε” followed a similar trend, and tan δ_μ_ had an increasing trend between 6 and 15 GHz, indicative of the strong magnetic loss capability at high frequencies. After doping rGO, tan δ_ε_ increased and tan δ_μ_ decreased, which indicates that the dielectric loss of HEA powders increased and the magnetic loss decreased.

### 3.4. Electromagnetic Wave Absorption Performance

**Figure 10:**
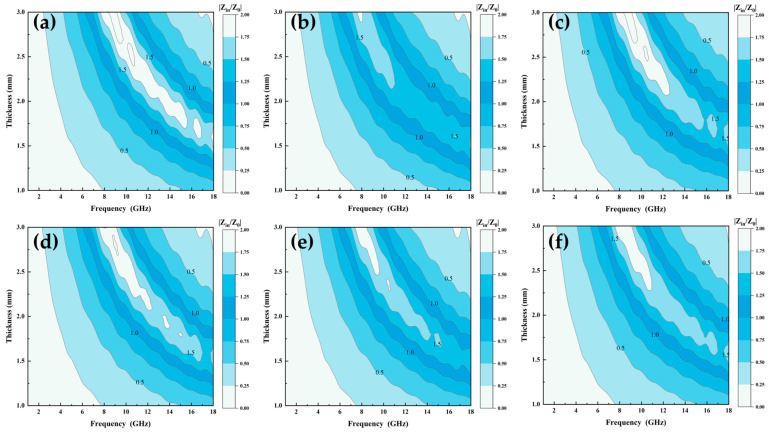
Impedance matching of Al_1.5_Co_4_Fe_2_Cr and Al_1.5_Co_4_Fe_2_Cr@rGO HEA powders: (**a**) A_300_, (**b**) A_350_, (**c**) A_400_, (**d**) B_300_, (**e**) B_350_, and (**f**) B_400_.

In addition to loss mechanism, impedance matching is the other main factor affecting the absorption performance of a material. Impedance matching can be calculated using the following equation [33]:

(6) $$z_{r}{=z}_{\mathrm{in}}{/z}_{0}=\sqrt{\frac{u_{r}}{\varepsilon_{r}}\cdot \tanh(j\frac{2\pi \mathrm{df}}{c}\sqrt{u_{r}\varepsilon_{r}})}$$

where Z_in_, Z_0_, d, c, u_r_, and ε_r_ are the impedance of the incident wave, the intrinsic impedance of free space, the thickness of the material, speed of light in free space, complex permittivity, and complex permeability of the material, respectively. A material with superior impedance-matching characteristics has a Z_in_/Z_0_ ratio closer to 1, which implies that electromagnetic wave incidents from the environment are not reflected from its surface, and most of the electromagnetic waves transmitted to the inside of the absorber are converted into absorbed internal energy [34]. Figure 10a–f show the impedance-matching diagrams of Al_1.5_Co_4_Fe_2_Cr and Al_1.5_Co_4_Fe_2_Cr@rGO, respectively. A two-dimension color-filled plot was used to describe the relationship between frequency, thickness, and Zr, where the darker color represented that the Zr value was 1. Compared with A_300_ and A_400_, B_300_ and B_400_ had a larger area of darker color in the 8–18 GHz range, meaning that their impedance matching was optimized. However, there was a small deterioration in the impedance matching of B_350_ compared with the A_350_ sample in Figure 10b, which was attributed to the fact that the doping of rGO did not improve the complex permittivity and complex permeability of A_350_. In addition, the impedance matching of HEA powders prepared at different rotational speeds was also significantly different, with the best impedance matching at a rotational speed of 350 rpm in comparison.

**Figure 11:**
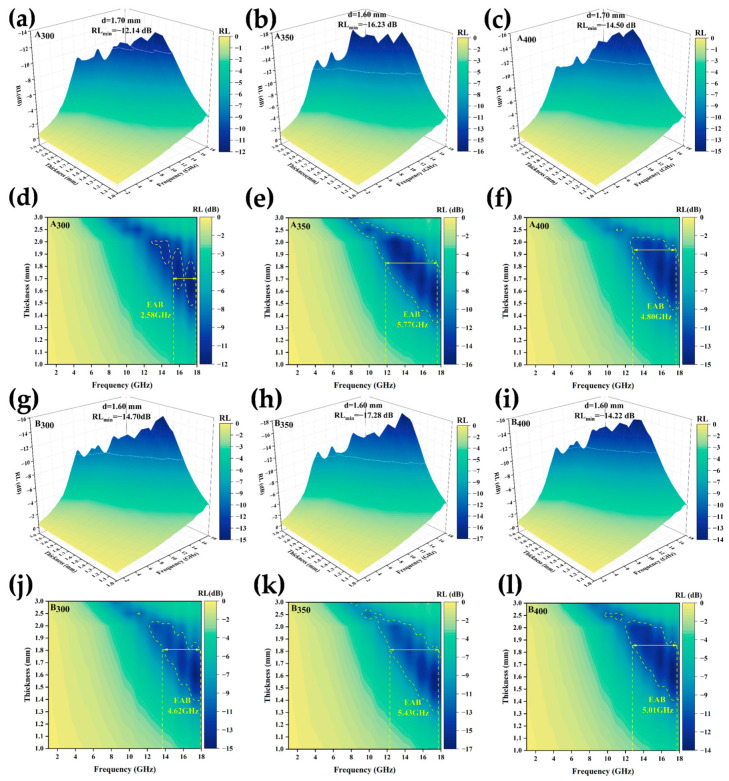
RL of Al_1.5_Co_4_Fe_2_Cr and Al_1.5_Co_4_Fe_2_Cr@rGO HEA powders prepared at different ball milling speeds: (**a**–**c**,**g**–**i**) 3D RL diagrams and (**d**–**f**,**j**–**l**) EAB diagrams: (**a**,**d**) A_300_, (**b**,**e**) A_350_, (**c**,**f**) A_400_, (**g**,**j**) B_300_, (**h**,**k**) B_350_, and (**i**,**l**) B_400_.

RL and EAB are two important parameters for measuring the absorption performance of a material. The lower the value of RL and the larger the value of EAB, the stronger the absorption ability and the wider the effective absorption frequency of the material. Generally, an RL less than −10 dB indicates that more than 90% of the incident microwaves can be absorbed, which is defined as the standard for effective microwave absorption [35]. According to the transmission line theory, the RL of the alloy powders can be calculated using the following formula [36,37,38,39]:

(7) $$\mathrm{RL}\left(\mathrm{dB}\right)=20\log\left|\frac{Z_{\mathrm{in}}\;{-\;Z}_{0}}{Z_{\mathrm{in}}{\;+\;Z}_{0}}\right|$$

where Z_in_ is the impedance of the material and Z_0_ is the intrinsic impedance in free space. Because both Z_in_ and Z_0_ are positive real numbers, the calculated RL must be negative. Therefore, the higher the absolute value of RL at a given frequency, the more effective the wave absorption performance [40]. The plotted 3D RL curves in Figure 11a–c,g–i show that the response frequency was concentrated in the medium and high-frequency ranges. The two best-performing samples (A_350_ and B_350_) had a peak absorption matching thickness of 1.6 mm and RL_min_ of −16.23 dB and −17.28 dB, respectively, and the absorption peaks shifted toward the low-frequency region as the sample thickness increased. The addition of defects introduced by rGO promoted the absorption of electromagnetic waves to a certain extent; at the same time, the layered rGO structure dispersed inside the absorber enhanced the interfacial reflection of electromagnetic waves, such that the RL_min_ of the alloy powder samples prepared at speeds of 300 and 350 rpm was slightly increased. However, as shown in Figure 11c,f, the RL_min_ and EAB of the alloy powders decreased at speeds up to 400 rpm. The main reasons for this were that faster milling speeds transferred higher kinetic energy to the powders, which introduced a variety of defects and impurities, and excessive defects had a negative impact on the magnetic properties of the material. On the other hand, the high rotational speed may have led to the destruction of the interlayer structure of rGO, which could not be uniformly dispersed in the HEAs, resulting in the reflection and scattering of electromagnetic waves, and thus leading to the reduction of the wave absorbing properties of A_400_ and B_400_. The curves in Figure 11(d–f, j–l) show the electromagnetic wave absorption frequency bandwidth of the powder samples. Similarly, the A_350_ and B_350_ alloy powders prepared at a speed of 350 rpm had the best EAB, reaching 5.77 GHz and 5.43 GHz, respectively. The addition of rGO increased the EAB of the sample at 300 and 400 rpm, however, it slightly decreased at 350 rpm. This may have been due to the fact that the B_350_ powders obtained a larger coercivity after adding rGO ball milling, which affected the complex permeability, and the impedance matching became poor, resulting in a narrower EAB.

**Figure 12:**
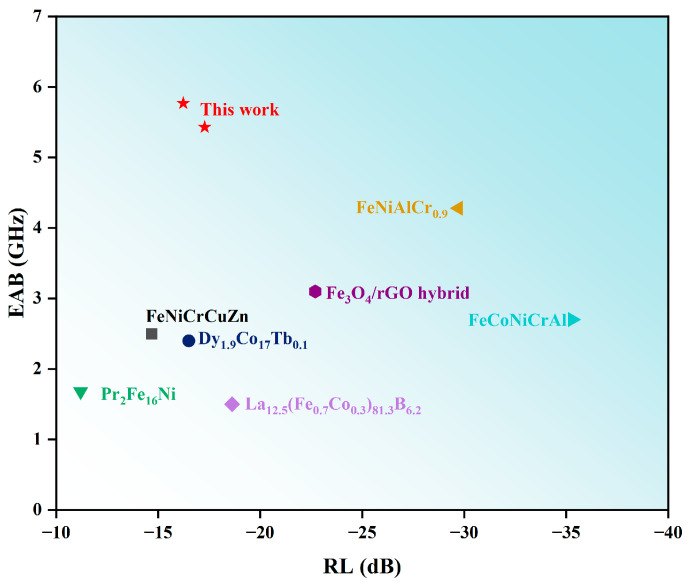
Comparative chart of the comprehensive performance for representative absorbers [41,42,43,44,45,46,47].

Al_1.5_Co_4_Fe_2_Cr and Al_1.5_Co_4_Fe_2_Cr@rGO were compared to other types of alloy microwave absorbing materials, and the comparison results are shown in Figure 12. As shown in Figure 12, Al_1.5_Co_4_Fe_2_Cr and Al_1.5_Co_4_Fe_2_Cr@rGO possessed excellent electromagnetic wave absorption, and the EAB reached a high position.

**Figure 13:**
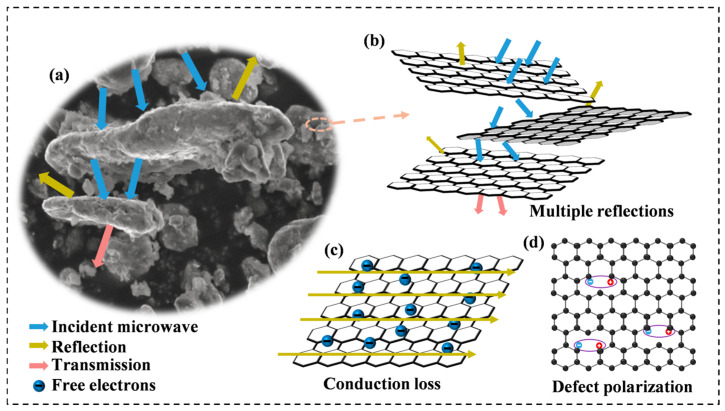
(**a**) Illustration of the interaction process between microwaves and the absorbent, (**b**) demonstration of reflection behavior of microwave on rGO surface, (**c**) conduction loss, and (**d**) defect polarization.

To fully understand the effect of rGO on the electromagnetic wave absorption process, the dissipation mechanism of electromagnetic waves in Al_1.5_Co_4_Fe_2_Cr@rGO HEA was analyzed in detail. Incident electromagnetic waves would be reflected, absorbed, or transmitted when the electromagnetic waves interacted with the absorbent, as shown in Figure 13a. Due to the irregular distribution of the multilayer rGO, the electromagnetic waves were reflected at multiple interfaces within the material, the propagation paths became more complex, and the electromagnetic waves would interact more with molecules and atoms in the wave absorbing material, thus increasing the energy loss and absorption. (Figure 13b). Although most of the electromagnetic waves were absorbed, a small portion of the electromagnetic waves was still reflected and scattered, and, when the rotational speed was increased, the interlayer structure of rGO was changed and the uniformity of distribution deteriorated, which narrowed the effective bandwidth and reduced the RL of the samples. On the other hand, the conduction loss occurring at the rGO surface also contributed to the dielectric loss of the absorber (Figure 13c) [48], and the enhancement of the complex dielectric constant by adding rGO also played an important role in optimizing the impedance-matching characteristics [49]. Apart from this, since the defect-excited polarization mainly originated from the defect sites in the absorber, the defects introduced by rGO could effectively promote defect polarization (Figure 13d). These defects were highly susceptible to relaxation behavior under the influence of an electromagnetic field, and the concentration of defects was positively correlated with the relaxation strength. These loss behaviors were believed to have contributed to the enhanced microwave absorption of the Al_1.5_Co_4_Fe_2_Cr@rGO HEA powders.

## 4. Conclusions

Al_1.5_Co_4_Fe_2_Cr and Al_1.5_Co_4_Fe_2_Cr@rGO alloy powder samples were prepared by an MA method at different ball milling speeds. The effects of the milling speed and the introduction of rGO on the electromagnetic properties and electromagnetic wave absorption performance of the alloy powders were investigated. It was important to keep the balance between the composite permeability and the composite dielectric constant of the material for improving the microwave absorption performance. The addition of rGO reduced the difference between the values of these properties, optimized the impedance matching characteristics, and increased the EAB of the B_300_ sample by 2.04 GHz compared with the A_300_ sample, as well as the RL_min_ by 2.56 dB. The Al_1.5_Co_4_Fe_2_Cr and Al_1.5_Co_4_Fe_2_Cr@rGO HEA powders prepared at 350rpm had the best performance, with RL_min_ of 16.23 and 17.28 dB at 17.3 GHz and EAB of 5.77 and 5.43 GHz at 1.6 mm, respectively, covering the whole Ku band. Increasing the ball milling speed exerted a larger force on the alloy powders and produced more lattice defects and impurities, and also destroyed the interlayer structure of rGO, all of which affected the magnetic properties of the alloy powders, most noticeably after the addition of rGO. This was the main reason for the reduced wave absorbing properties of A_400_ and B_400_. But, in general, rGO could improve the complex dielectric constant of the Al_1.5_Co_4_Fe_2_Cr HEA powders and served to enhance and optimize the impedance matching and wave absorption properties, and the rGO lamellae distributed within the absorber would also enhance the interfacial reflection behavior of electromagnetic waves; all these properties promoted the absorption of electromagnetic waves by HEA wave absorbers.

## Figures and Tables

**Table 1:** Chemical composition of samples (wt. %).

	Al	Co	Fe	Cr	C	O
A_300_	8.28	50.98	24.96	12.40	1.36	2.02
A_350_	7.94	51.32	25.03	11.39	1.89	2.43
A_400_	7.21	49.26	26.21	12.68	2.04	2.60
B_300_	7.86	48.57	25.44	12.75	2.81	2.57
B_350_	8.43	50.20	24.09	11.96	2.97	2.35
B_400_	7.57	49.54	26.33	10.88	2.85	2.83

## Data Availability

The data that support the findings of this study are available from the corresponding author upon reasonable request.
